# Molecular Hazard Identification of Non-O157 Shiga Toxin-Producing *Escherichia coli* (STEC)

**DOI:** 10.1371/journal.pone.0120353

**Published:** 2015-03-19

**Authors:** Eelco Franz, Angela H. A. M. van Hoek, Mark Wuite, Fimme J. van der Wal, Albert G. de Boer, EI Bouw, Henk J. M. Aarts

**Affiliations:** 1 National Institute for Public Health and the Environment (RIVM), Centre for Infectious Disease Control, Bilthoven, the Netherlands; 2 Central Veterinary Institute, Wageningen University & Research Centre, Lelystad, the Netherlands; Beijing Institute of Microbiology and Epidemiology, CHINA

## Abstract

The complexity regarding Shiga toxin-producing *Escherichia coli* (STEC) in food safety enforcement as well as clinical care primarily relates to the current inability of an accurate risk assessment of individual strains due to the large variety in serotype and genetic content associated with (severe) disease. In order to classify the clinical and/or epidemic potential of a STEC isolate at an early stage it is crucial to identify virulence characteristics of putative pathogens from genomic information, which is referred to as ‘predictive hazard identification’. This study aimed at identifying associations between virulence factors, phylogenetic groups, isolation sources and seropathotypes. Most non-O157 STEC in the Netherlands belong to phylogroup B1 and are characterized by the presence of *ehxA*, *iha* and *stx*_2_, but absence of *eae*. The large variability in the number of virulence factors present among serogroups and seropathotypes demonstrated that this was merely indicative for the virulence potential. While all the virulence gene associations have been worked out, it appeared that there is no specific pattern that would unambiguously enable hazard identification for an STEC strain. However, the strong correlations between virulence factors indicate that these arrays are not a random collection but are rather specific sets. Especially the presence of *eae* was strongly correlated to the presence of many of the other virulence genes, including all non-LEE encoded effectors. Different *stx*-subtypes were associated with different virulence profiles. The factors *ehxA* and *ureC* were significantly associated with HUS-associated strains (HAS) and not correlated to the presence of *eae*. This indicates their candidacy as important pathogenicity markers next to *eae* and *stx*_2a_.

## Introduction

Shiga toxin producing *Escherichia coli* (STEC) are potential lethal zoonotic pathogens with a clinical spectrum including diarrhea, hemorrhagic colitis, and the hemolytic uremic syndrome (HUS) [1]. STEC is of significant public health concern given the potential for foodborne outbreaks and their strong association with HUS, which is the leading cause of acute renal failure in children. The most common STEC serotype associated with human disease is O157:H7, but there is a growing recognition of over a hundred non-O157 STEC serotypes that also may result in human illness [2–4]. Some of these non-O157 STEC strains cause outbreaks and severe disease, whereas others are associated with only mild sequela or with no human disease at all [5]. This observation resulted in the development of the STEC seropathotype (SPT) classification, which is based on the serotype association with human epidemics and HUS [6]. Serotypes responsible for haemorrhagic colitis (HC) and haemolytic uraemic syndrome (HUS), O157:H7 and O157:NM, were assigned to seropathotype A. Seropathotype B strains (O26:H11, O103:H2, O111:NM, O121:H19 and O145:NM) have a strong association with outbreaks and HUS, but less commonly than those of seropathotype A. Seropathotype C serotypes (O91:H21, O104:H21, O113:H21, O5:NM, O121:NM and O165:H25) aree associated with sporadic HUS cases but not with epidemics. Seropathotype D serotypes are associated with diarrhea but not with HUS and/or outbreaks. Seropathotype E serotypes comprise STEC that had never been associated with human disease and had been isolated only from animals. Following the scientific opinion of the European Food Safety Authority (EFSA) [7] an alternative grouping (modified SPT) was used in which all serotypes associated with severe disease (HUS) were categorized as seropathotype group ”haemolytic uraemic syndrome (HUS)-associated serotype(s)” or HAS (Karmali groups A, B and C). Isolates not associated with HUS were grouped as SPT-D or SPT-E following the same criteria as the Karmali classification.

The EFSA network recently concluded that the seropathotype classification does not define pathogenic STEC nor does it provide an exhaustive list of pathogenic serotypes [7]. This relates primarily to the complexity of designating individual strains as pathogens due to the large variety in serotype and genetic content associated with (severe) disease. There is mounting evidence suggesting that the pathogenesis of STEC infection involves many additional virulence factors besides the well-known Shiga toxins and the locus of enterocyte effacement (LEE), including effector molecules encoded on pathogenicity islands (PAIs) outside the LEE [8,9]. In addition, there are considerable differences in geographic distribution of human pathogenic STEC serogroups [10]. Finally, although informative as an *ex post facto* determinant of virulence potential, the dynamic nature of STEC virulence in time and place exposes a limitation of SPT classification as a predictive indicator of microbial risk [5].

Phylogenetic analyses have shown that most *E*. *coli* strains belonged to four main phylogenetic groups, A, B1, B2, and D [11]. Whereas most commensal and diarrheogenic strains assemble in groups A and B1, extraintestinal *E*. *coli* strains belong mainly to group B2 and group D. STEC strains were found to fall into phylogenetic groups A, B1, and D [12]. However, there is a lack of knowledge on the phylogenetic distribution of the virulence factors of STEC isolates.

The serogroups most frequently associated with severe human disease in the EU, in particular HUS, are O157, O26, O111, O103, and O145 [7]. Epidemiological surveillance in the Netherlands in 2012 revealed that of all confirmed STEC infections 30% was due to serogroup O157 and 70% due to non-O157 serogroups [13]. Over the period 2007–2012 the most reported non-O157 serotypes were O26 (12%), O63 (10%), O91 (9%), O113 (6%), O103 (5%) and O146 (4%) [13].

The goal of this study was to investigate the distribution of known virulence factors among clinical, food and animal STEC isolates from the Netherlands. More specific, the research aimed at identifying associations between virulence factors and phylogenetic groups, isolation sources, seropathotypes, serogroups, intimin presence/absence, type of Shiga toxin, and the *rpoS* genotype. The results are discussed in relation to the epidemiology of STEC in the Netherlands.

## Materials and Methods

### Isolates and growth conditions

A set of 209 STEC non-O157 isolates (23 animal, 57 meat and 129 human clinical isolates) was obtained from the collection of the Food and Consumer Product Safety Authority (Wageningen, the Netherlands) and the National Institute for Public Health and The Environment (Bilthoven, the Netherlands). The animal isolates (all bovine) were isolated from 2002 to 2009 during national surveys and were maintained in Microbank vials (bioTRADING, Mijdrecht, the Netherlands). The strains from meat (different types of meat from various food animals) were isolated during national surveys by the Food and Consumer Product Safety Authority in the Netherlands 2005–2010. The clinical human isolates were strains isolated from patients with STEC symptoms and sent in by hospitals for confirmation in the period 2006 to 2010. All clinical human isolates were maintained at room temperature in Mueller-Hinton agar.

Isolates were propagated on blood agar or nutrient agar (Oxoid) and DNA was extracted using Chelex-100 (Bio-Rad Laboratories B.V., Veenendaal, the Netherlands) resin-based technique. One colony of each isolate was transferred into 300 μl 10% Chelex-100 solution, which was subsequently heated for 5 min at 56°C to resuspend the cells. The tubes were briefly cooled at room temperature and mixed for 15 seconds before heating for 15 min at 98–99°C for lysis of the bacteria. After cooling at room temperature, the lysates were centrifuged for 5 min. at 13,000 rpm and up to 200 μl of the supernatant was transferred to a clean tube and stored at -20°C.

### Seropathotype grouping and genetic profiling

Isolates were grouped according to the Karmali seropathotype classification [6]. Ranking was done based on the clinical symptoms caused by the Dutch patient isolates, the German HUS-associated EHEC (HUSEC) reference strain collection [14], and data on clinical outcome of confirmed STEC cases in humans in the EU from the European Surveillance System (TESSy) (2007–2010) as provided by the European Centre for Disease Prevention and Control (ECDC) [7].

PCR was used to screen isolates for the presence of 40 virulence markers and determination of the phylogenetic group. Primers and probes used in this study are displayed in Table 1. Conventional PCR tests for *adfO*, *astA*, *ckf*, *efaI*, *ent*/*espL*2, *etpD*, *iha*, *iha*_homologue, *saa*, *stx*_2a_, *stx*_2b_, *stx*_2c_, *stx*_2d_, *stx*_2dact_, *stx*_2e_, *stx*_2f_, *stx*_2g_, *subA*, *toxB* and *ureC* were performed on a Thermo Hybaid PCR Express Thermal Cycler (Hybaid Limited, Ashford, Middlesex, UK) using iQ Supermix (Bio-Rad Laboratories B.V., Veenendaal, the Netherlands) and 0.2 μM of each primer at an annealing temperature as indicated in Table 1. PCR products were visualized on a 1.5% or 2% (depending on the length of the fragment (see Table 1)) agarose gel. Real-time PCR tests for *eae*, *ehxA*, *stx*_1_, *stx*_2_ and *terB* were performed on an iQ5 Thermal Cycler using iQ Supermix (Bio-Rad Laboratories B.V., Veenendaal, the Netherlands) 0.2 μM of each forward and reverse primer and 0.4 μM of the probe at the temperature as indicated in Table 1.

**Table 1:** Primers and probes used for gene-content analysis in non-O157 STEC.

Target	Genetic support	Encoded protein or family effector	Primer sequences (5’-3’)	Annealing temperature (°C)	Productsize (bp)	Reference
*adfO*	O-Island 57	Adhesin	Forward: TGGTGGCCCGCATACAGCReverse: TGCCCAGTCAGCCCAGGTTA	58	501	[47]
*astA*	Plasmid and chromosome	Heat-stable enterotoxin EAST1	Forward: CCATCAACACAGTATATCCGAReverse: GGTCGCGAGTGACGGCTTTGT	60	111	[48]
*chuA* ^a^	Chromosome	Heme/hemoglobin receptor	Forward: GACGAACCAACGGTCAGGATReverse: TGCCGCCAGTACCAAAGACA	60	279	[16]
*ckf*	O-Island 57	Putative killer protein	Forward: ATGCTCGTCACATATAGATTGReverse: GTTCGTAAGCTGTGAAGACA	58	201	[47]
*eae*	LEE	Intimin adhesin	Forward: CATTGATCAGGATTTTTCTGGTGATAReverse: CTCATGCGGAAATAGCCGTTAProbe: FAM-ATAGTCTCGCCAGTATTCGCCACCAATACC-BHQ1	60	102	[49]
*efa1*	O-Island 122	EHEC factor for adherence	Forward: CTCCCAGAGATAATTTTGAGGReverse: CAACTGTATGCGAATAGTACTC	60	504	[6]
*ehxA*	pO157	Enterohemolysin	Forward: ATCATGTTTTCCGCCAATGReverse: ATCATGTTTTCCGCCAATGProbe: FAM-CGTGATTTTGAATTCAGAACCGGTGG-BHQ1	55	126	[34]
*ent/espL2*	O-Island 122	Microcolony formation and F-actin aggregation	Forward: GAATAACAATCACTCCTCACCReverse: TTACAGTGCCCGATTACG	55	233	[8]
*etpD*	pO157	Type-II effector	Forward: TCGTCAGGAGGATGTTCAGReverse: CGACTGCACCTGTTCCTGATTA	58	1,063	[50]
*iha*	O-Island 43 & O-Island 48	Iron regulated adhesin	Forward: TGTATGGCTCTGATGCGATG	60	1,218	[51]
			Reverse: AGGTGTAATTCAGTGACAGCG			This study
*iha_*homologue	O-Island 43 & O-Island 48	Iron regulated adhesin	Forward: TGTATGGCTCTGATGCGATGReverse: GTCCAGGCATCGGTCACG	60	867	[51]This study
*nleA*	O-Island 71	Disruption tight junctions and protein trafficking	Forward: ATGAACATTCAACCGACCATACReverse: GACTCTTGTTTCTTGGATTATATCAAA	55	1,296	[8]
*nleB*	O-Island 122	Immunomodulation	Forward: GGAAGTTTGTTTACAGAGACG Reverse: AAAATGCCGCTTGATACC	55	297	[8]
*nleB2*	O-Island 36	Non-LEE encoded type III effector	Forward: GTTAATACTAAGCAGCATCCReverse: CCATATCAAGATAGATACACC	52	475	[8]
*nleC*	O-Island 36	Immunomodulation, zinc-metalloprotease	Forward: ACAGTCCAACTTCAACTTTTCCReverse: ATCGTACCCAGCCTTTCG	55	777	[8]
*nleD*	O-Island 36	Immunomodulation, zinc-metalloprotease	Forward: GGTATTACATCAGTCATCAAGGReverse: TTGTGGAAAACATGGAGC	55	426	[8]
*nleE*	O-Island 122	Immunomodulation	Forward: GTATAACCAGAGGAGTAGCReverse: GATCTTACAACAAATGTCC	52	260	[8]
*nleF*	O-Island 71	Disruption protein trafficking	Forward: ATGTTACCAACAAGTGGTTCTTCReverse: ATCCACATTGTAAAGATCCTTTGTT	55	567	[8]
*nleG*	O-Island 71	Ubiquitin ligase	Forward: ATGTTATCGCCCTCTTCTATAAATReverse: ACTTAATACTACACTAATAAGATCCA	55	902	[8]
*nleG2–1*	O-Island 71	Ubiquitin ligase	Forward: ACCAGAAACCTGACTTCGReverse: CAGCATCTTCATATACTACAGC	55	406	[8]
*nleG2–3*	O-Island 57	Ubiquitin ligase	Forward: GGATGGAACCATACCTGGReverse: CGCAATCAATTGCTAATGC	56	551	[8]
*nleG5–2*	O-Island 57	Ubiquitin ligase	Forward: TGGAGGCTTTACGTCATGTCGReverse: CCGGAACAAAGGGTTCACG	55	504	[8]
*nleG6–2*	O-Island 57	Ubiquitin ligase	Forward: CGGGTCAGTGGATGATATGAGCReverse: AAGTAGCATCTAGCGGTCGAGG	55	424	[8]
*nleG9*	O-Island 71	Ubiquitin ligase	Forward: GTTCGTGCCCGAATTGTAGCReverse: CACCAACCAAACGAGAAAATG	55	409	[8]
*nleH1–1*	O-Island 36	Immunomodulation	Forward: GTTACCACCTTAAGTATCCReverse: GTTTCTCATGAACACTCC	55	456	[8]
*nleH1–2*	O-Island 71	Immunomodulation	Forward: AACGCCTTATATTTTACCReverse: AGCACAATTATCTCTTCC	52	589	[8]
*rpoS*	Chromosome	RNA polymerase, sigma S	Forward: CTTGCATTTTGAAATTCGTTACReverse: GATGATGAACACATAGGATGCReverse: GCCAGCCTCGCTTGAGACSequencing: CCTTATGAGTCAGAATACGCSequencing: CTCTGCTTCATATCGTCATC	56	1,2581,163	[15]This study[15]
*saa*	pO113	STEC autoagglunating adhesin	Forward: CGTGATGAACAGGCTATTGCReverse: ATGGACATGCCTGTGGCAAC	60	119	[52]
*stx*_1_	Chromosome	Shiga toxin	Forward: TTTGTYACTGTSACAGCWGAAGCYTTACGReverse: CCCCAGTTCARWGTRAGRTCMACRTCProbe: FAM-CTGGATGATCTCAGTGGGCGTTCTTATGTAA-BHQ1	60	131	[53]
*stx*_2_	Chromosome	Shiga toxin	Forward: TTTGTYACTGTSACAGCWGAAGCYTTACGReverse: CCCCAGTTCARWGTRAGRTCMACRTCProbe: FAM-TCGTCAGGCACTGTCTGAAACTGCTCC-BHQ1	60	128	[53]
*stx*_2a_	Chromosome	Shiga toxin	Forward: GATACTGRGBACTGTGGCCReverse: CCGKCAACCTTCACTGTAAATGTGReverse: GCCACCTTCACTGTGAATGTG	65	349347	[54]
*stx*_2b_	Chromosome	Shiga toxin	Forward: AAATATGAAGAAGATATTTGTAGReverse: CAGCAAATCCTGAACCTGACG	65	251	[54]
*stx*_2c_	Chromosome	Shiga toxin	Forward: GAAAGTCACAGTTTTTATATACAACGReverse: CCGGCCACYTTTACTGTGAATGTA	65	177	[54]
*stx*_2d_	Chromosome	Shiga toxin	Forward: AAARTCACAGTCTTTATATACAACGGGReverse: TTYCCGGCCACTTTTACTGTG	65	179	[54]
*stx*_2dact_	Chromosome	Shiga toxin	Forward: AAARTCACAGTCTTTATATACAACGGGReverse: GCCTGATGCACAGGTACTGGAC	65	280	[54]
*stx*_2e_	Chromosome	Shiga toxin	Forward: CGGAGTATCGGGGAGAGGCReverse: CTTCCTGACACCTTCACAGTAAA	65	411	[54]
*stx*_2f_	Chromosome	Shiga toxin	Forward: TGGGCGTCATTCACTGGTTGReverse: TAATGGCCGCCCTGTCTCC	65	424	[54]
*stx*_2g_	Chromosome	Shiga toxin	Forward: CACCGGGTAGTTATATTTCTGTGGATReverse: GATGGCAATTCAGAATAACCGCT	65	573	[54]
*subA*	pO113	Subtilase cytotoxin	Forward: TATGGCTTCCCTCATTGCReverse: TATAGCTGTTGCTTCTGACG	54	556	[55]
*terB*	OI-43 & OI-48	Tellurite resistance cluster	Forward: GCCAGGTTGGCCGTTTCReverse: CCGTCACTCGATACGGCAATProbe: FAM-AAAACAAAAAATTCATGCAGGGCACCG-BHQ1	55	82	[31]
*toxB*	pO157	Homolog of *efa1*, adhesin	Forward: CAACAGCCCCTTCATTCCATTCReverse: TTGCCACATTGCTAAGATAACG	58	555	This study
TspE4.C2^a^	chromosome	Esterase-lipase protein	Forward: GAGTAATGTCGGGGCATTCAReverse: CGCGCCAACAAAGTATTACG	60	152	[16]
*ureC*	OI-43 & OI-48	Urease-associated protein	Forward: TCTAACGCCACAACCTGTACReverse: GAGGAAGGCAGAATATTGGG	60	397	[56]
*yjaA* ^a^	chromosome	Unknown	Forward: TGAAGTGTCAGGAGACGCTGReverse: ATGGAGAATGCGTTCCTCAAC	60	211	[16]

Note:

^a^ These targets were used in a multiplex format in the phylogenetic group PCR.

PCRs for the non-LEE encoded effectors *(nle*) and *ent*/*espL2* were performed as described in Coombes *et al*. [8]. Except for *nleA*, the forward primer of each primer set was fluorescently labelled with FAM, VIC, NED, or PET. Amplicons were generated essentially as described and pooled in five sets, resulting in distinctive combinations of fragment size and fluorescent label. Fluorescently labelled fragments were analyzed on a capillary sequencer (3130 Genetic Analyzer; Applied Biosystems) in the presence of an internal marker (GeneScan size standard; Applied Biosystems). The GeneScan 600 LIZ size standard was used for pooled amplicons smaller than 600 bp (pool 1: *ent/espL2*, *nleG2-1*, *nleB2*, *nleH1-2*; pool 2: *nleE*, *nleG9*, *nleH1-1*, *nleG2-3*; pool 3: *nleB*, *nleG6-2*, *nleG5-2*; pool 4: *nleD* and *nleF*). The GeneScan 1200 LIZ size standard was used for pooled PCR products between 600 and 1,200 bp (*nleC* and *nleG*). Fragments larger than 1,200 bp (*nleA*; 1,296 bp) were analyzed by agarose gel electrophoresis. Raw data were analyzed using BioNumerics 6.1 (Applied Maths) to determine fragment sizes.PCR amplification and sequencing of the *rpoS* gene was performed as described earlier [15], but for several isolates, an alternative reverse primer was used to obtain the complete open reading frame (Table 1).

The phylogenetic group PCR amplifying parts of *chuA*, TspE4.C2 and *yjaA* was carried out in a multiplex format using the Qiagen multiplex PCR mix and 0.2 μM of each primer at an annealing temperature of 60°C (Table 1) [16].

### Data analysis

Differences in frequencies of genetic markers (denoted in binary values 0 and 1) between groups and associations between markers were evaluated using the Chi-square test with a significance level of 0.05 (IBM SPSS Statistics version 19).

## Results

Isolate characteristics and the PCR results of all strains used can be accessed in S1 Table.

### Distribution of virulence factors over serogroups

**Fig 1:**
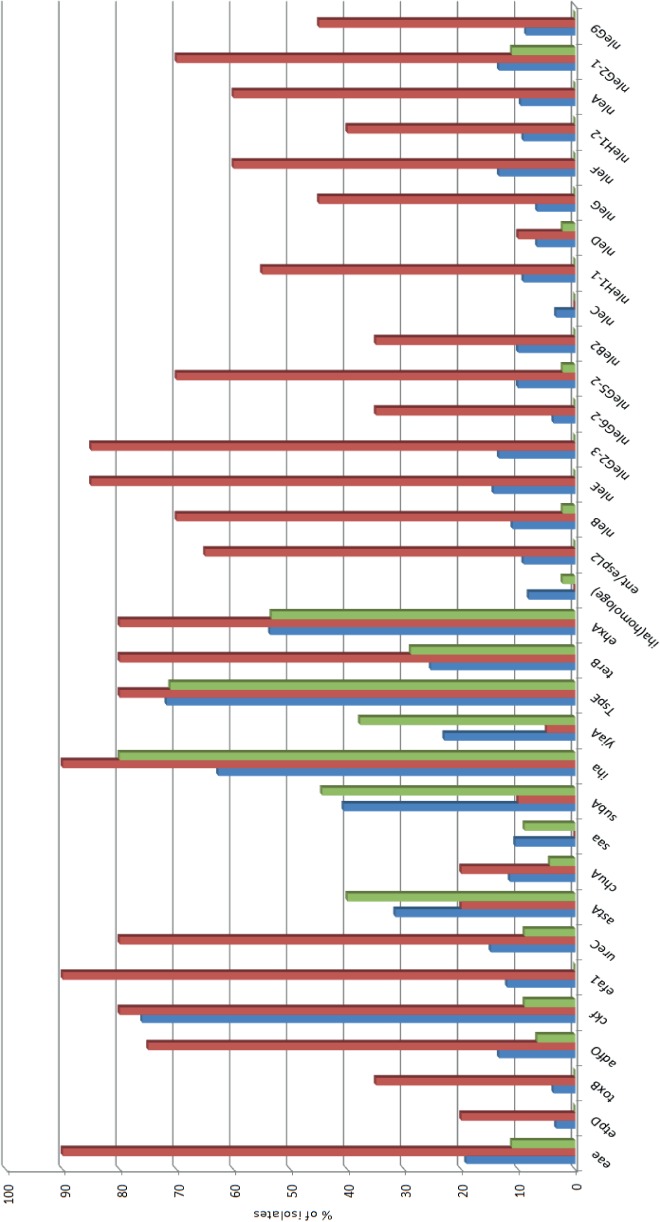
Prevalence of virulence genes among all STEC included in this study (blue bars, n = 209), the top 4 most important non-O157 serogroups (O26, O103, O111 and O145) in the European Union present in our dataset (red bars, n = 20), and the top 4 most important non-O157 serogroups (excluding the top 4 EU serogroups; O63, O91, O113 and O146) in the Netherlands present in our dataset (green bars, n = 45).

**Fig 2:**
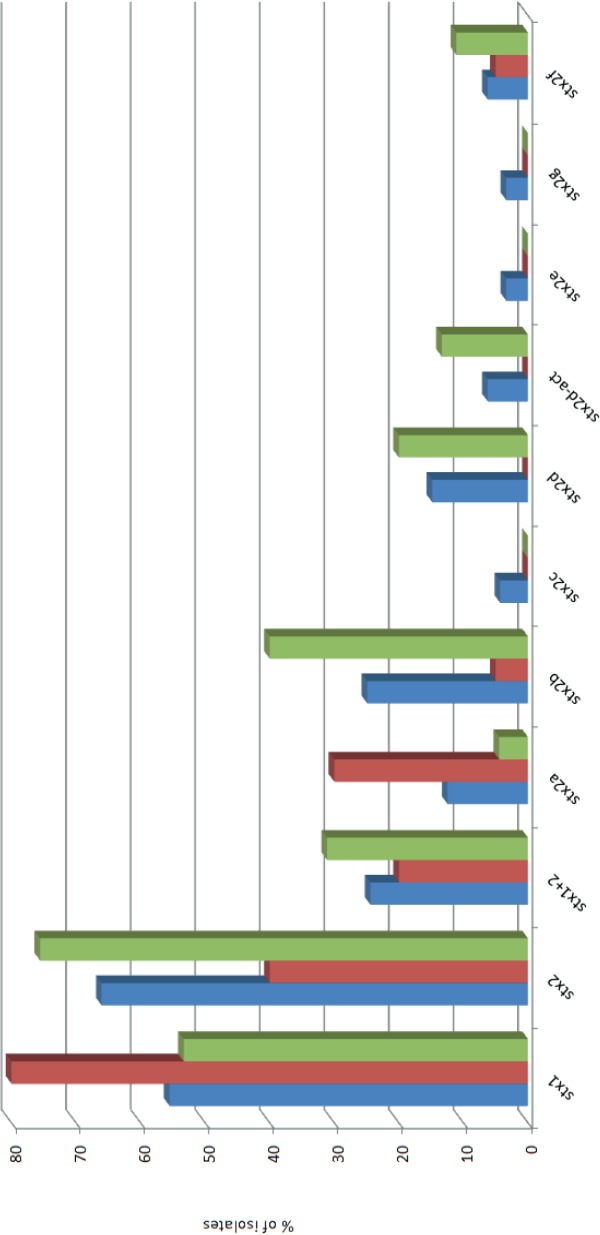
Distribution of Shiga toxin subtypes among all STEC isolates included in this study (blue bars, n = 209), the top 4 most important non-O157 serogroups (O26, O103, O111 and O145) in the European Union present in our dataset (red bars, n = 20), and the top 4 most important non-O157 serogroups (excluding the top 4 EU serogroups; O63, O91, O113 and O146) in the Netherlands present in our dataset (green bars, n = 45.

Overall, the vast majority of STEC strains included in this study were *eae*-negative (169/209 = 80.9%) (Fig. 1). A relatively high overall prevalence of *stx*_1_(78%) was observed (Fig. 2). Interestingly, *stx*_2_ showed a relatively low prevalence (39%) in the top-EU serotypes (O26, O103, O111 and O145) isolated in the Netherlands while the prevalence of s*tx*_1_ was relatively high among these serogroups. The genes *stx*_2_ (more specific: *stx*_2b_ and *stx*_2d_) and *subA* occurred at significant higher frequency (P<0.05) among the most frequently isolated clinical serogroups in the Netherlands (O63, O91, O113 and O146; excluding the top 4 EU serogroups) compared to the top 4 EU serogroups (O26, O103, O111 and O145). The following genes occurred at significant lower frequency among the top 4 Dutch serogroups compared to the top 4 EU serogroups (in random order): *eae*, *stx*_2a_, *etpD*, *toxB*, *adfO*, *cfk*, *efa*. *ureC*, *terB*, *ehxA*, *ent/espL2*, *nleB*, *nleE*, *nleG23*, *nleG62*, *nleG52*, *nleB2*, *nleH11*, *nleG*, *nleF*, *nleH12*, *nleA*, *nleG21 and nleG9*. In addition, the top Dutch serogroups were significantly stronger associated with phylogroup A (12/45 versus 0/20) (P = 0.021) and showed significantly less total number of virulence genes (median of 6 versus 11) (P = 0.002).

The number of virulence genes present in the top 4 EU serogroups showed considerable variation: O26 (n = 7) average 20.7 (14–25), O111 (n = 5) average 17.7 (7–23), O103 (n = 3) average 17.3 (7–24), O145 (n = 5) average 16.4 (7–19), and O121 (n = 2) 5. Some isolates classified as Karmali SPT-C-E or modified SPT-D and E contained relatively high numbers of virulence genes: O84:H- (17, n = 1), O5:H- (18, n = 1), O165: H- (17, n = 1), O6:H25 (22, n = 1); O80: H- (17, n = 1), O55:H7 (20, n = 1) and O177: H- (18, n = 2).

### Association between *stx*-type and other virulence genes

**Fig 3:**
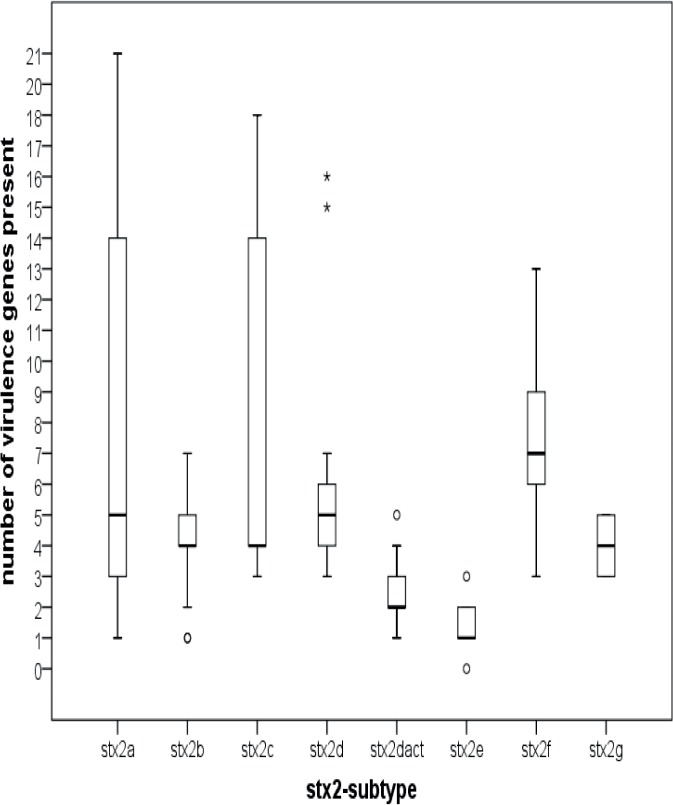
Boxplots of the number of virulence markers present in isolates with different *stx*-subtypes. Solid horizontal line represents the median, the box represents the 25%-75% quartile range, the stems represent the minimum and maximum values, asterisks represent outliers.

On average, isolates with *stx*_2a_, *stx*_2c_ and s*tx*_2f_ showed higher number of virulence genes compared to isolate with the other *stx*-subtypes present (Fig. 3). Subtype *stx*_2a_ and *stx*_2f_ were significantly (Chi-square P<0.05) associated with *eae* (Table 2). Especially *stx*_2a_ was associated with a large number of *nle*-genes. In contrast, *stx*_2d_ and *stx*_2dact_ were negatively associated with *eae*, and s*tx*_2e_ showed no specific positively associated virulence factors. The *stx*_2f_ isolates showed significant (P<0.001) positive associations with (in alphabetic order): *adfO*, *astA*, *cfk*, *eae*, *nleB2*, *nleD*, and *nleF*. Negative associations were observed with *ehxA*, *iha*, *nleG21*, *stx*_1_, *subA and terB* (Table 2).

**Table 2:** Significant (Chi-square P<0.05) positive and negative associated virulence markers with different *stx*-subtypes.

*stx*-subtype	Positive associated virulence genes	Negative associated virulence genes
*stx*_2a_	*eae*, *efa1*, *ehxA*, *ent/espL2*, *nleB*, *nleB2*, *nleC*, *nleE*, *nleF*, *nleG2–3*, *nleG5–2*, *nleG9*, *nleH1–1*, *nleH1–2*, *saa*, *subA*	-
*stx*_2b_	*iha*, *stx*_1_, *subA*	*adfO*, *cfk*, *efa1*, *ent/espL2*, *nleA*, *nleB*, *nleB2*, *nleD*, *nleE*, *nleF*, *nleG*, *nleG2–3*, *nleG5–2*, *nleH1–1*, *nleH1–2*, *nleG2–1*, *nleG9*, *saa*, *stx*_1_
*stx*_2c_	*nleC*, *nleD*, *nleH1–1*, *nleH1–2*, *nleG9*, *saa*	*stx*_1_
*stx*_2d_	*iha*, *saa*	*adfO*, *eae*, *ehxA*, *stx*_1_, *subA*, *ureC*
*stx*_2dact_	*iha*, *saa*	*adfO*, *eae*, *ehxA*, *stx*_1_, *subA*, *ureC*
*stx*_2e_	-	*ehxA*, *iha*, *stx*_1_, *subA*
*stx*_2f_	*adfO*, *astA*, *cfk*, *eae*, *nleB2*, *nleD*, *nleF*, *nleG2–1*	*ehxA*, *iha*, *stx*_1_, *subA*, *terB*
*stx*_2g_	*astA*, *ureC*	*stx*_1_, *subA*

### Phylogenetic distribution of STEC and association with virulence genes

The majority (63.2%) of the STEC isolates included in this study was characterized as phylogenetic group (phylotype) B1, followed by A (20.1%), D (9.1%) and B2 (7.7%). The distribution of phylotypes was not significantly different among animal, meat and human isolates (χ2 = 3.87, P = 0.424). However, a trend was observed with relatively more A and B1 isolates among non-human isolates (90.0%) compared to human isolates (79.1%). In contrast, relatively more B2 and D isolate were observed among human isolates (20.9%) compared to non-human isolates (10.0%). A significant association between phylogenetic group and HAS was observed (χ2 = 10.68, P = 0.014), with no HAS among phylogenetic group A (n = 42) and B2 (n = 16). In contrast, 85.2% of the HAS belonged to phylogenetic group B1, and 14.8% to phylogenetic group D.

No difference was observed in the number of virulence genes present in isolates of different phylogenetic groups (P = 0.515). However, some genes differed significantly in frequency between different phylogenetic groups (Table 3). The *eae* gene was more likely to be associated with B2 and D isolates compared to A and B1 isolates. Shiga toxin subtype 2f showed a significant association with phylogenetic group B2. Isolates with *stx*_2e_ and *stx*_2g_ were significantly associated with phylogenetic group A. Similarly, *stx*_2f_, *adfO*, and *nleB2* were more likely to be associated with B2 and D isolates. In contrast, *stx*_2_, *iha*, and *ehxA* were more likely to be associated with A and B1 isolates.

**Table 3:** Phylogenetic distribution of virulence genes.

Phylogroup/Virulence gene	An = 42	B1n = 132	B2n = 16	Dn = 19	P-value	ORA+B1 vs. B2+D
*adfO*	7%	10%	44%	26%	0.003	0.19
*eae*	24%	15%	50%	26%	0.006	0.29
*ehxA*	43%	65%	6%	37%	<0.001	3.33
*iha*	38%	77%	31%	42%	<0.001	3.33
*nleB2*	14%	5%	25%	26%	0.004	0.20
*nleC*	12%	0%	19%	5%	0.002	n.s.
*nleD*	19%	2%	19%	5%	<0.001	n.s.
*stx*_1_	31%	68%	31%	42%	<0.001	n.s.
*stx*_2_	81%	62%	19%	58%	<0.001	3.33
*stx*_2f_	5%	2%	56%	0%	<0.001	0.07
*stx*_2g_	11%	2%	0%	0%	0.007	n.s.
*subA*	21%	51%	19%	32%	0.001	n.s.

Only virulence factors showing a P-value (from Chi-square test) <0.01 are shown (in alphabetic order). Percentages do not add up to 100% since fractions are per phylogroup.

### Virulence factors in relation to STEC seropathotype

**Fig 4:**
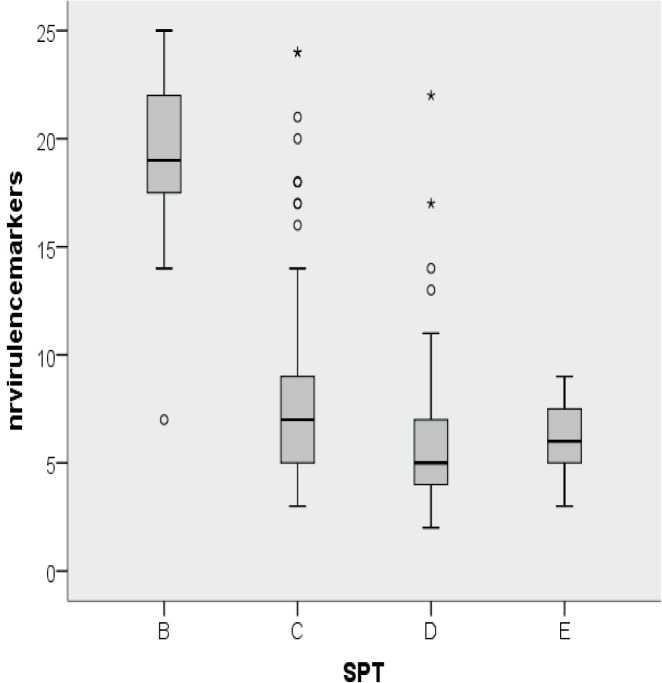
Boxplot of number of total number of virulence markers among seropathotypes according the classicial Karmali classification. Solid horizontal line represents the median, the box represents the 25%-75% quartile range, the stems represent the minimum and maximum values, asterisks represent outliers.

**Fig 5:**
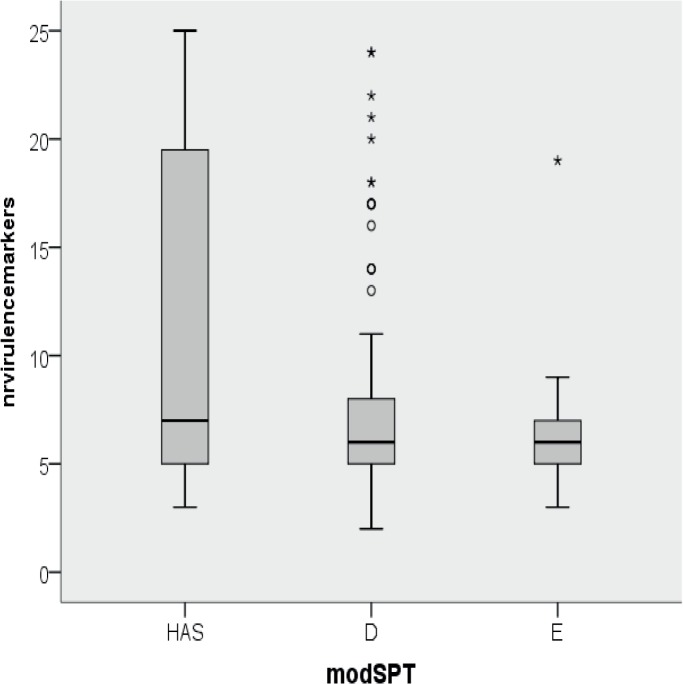
Boxplot of number of total number of virulence markers among seropathotypes according to the modified classification (modSPT). Solid horizontal line represents the median, the box represents the 25%-75% quartile range, the stems represent the minimum and maximum values, asterisks represent outliers.

The total number of markers present decreased progressively from the Karmali SPT-B to SPT-E (Fig. 4). SPT-B showed significant higher number of virulence genes (mean 18.9) than SPT-C (mean 8.3) (P<0.001), SPT-D (mean 6.0) (P<0.001) and SPT-E (mean 6.2) (P<0.001). SPT-C showed a significant higher mean number compared to SPT-D (P = 0.025) and SPT-E (P = 0.012). No difference was observed between SPT-D and SPT-E (P = 0.999). When considering the modified SPT classification, HAS isolated showed a significant higher number of markers (mean 9.3, median 7) (P<0.001) compared to non-HAS isolates (mean 6.1, median 6) (Fig. 5). The decrease in mean number of virulence genes from Karmali SPT-B to SPT-E and from HAS to SPT-E was primarily due to the decrease in amount of *nle*-genes.

### Virulence factors differentiating human versus non-human isolates

When considering all genetic markers investigated in this study there was no significant difference in the number of genes found present between non-human (mean 6.1) and human isolates (mean 7.0) (P = 0.142). Irrespective of serogroup and seropathotype, some genetic targets were found at a significantly different frequency among isolates of human and non-human origin (Table 4). Highly significant (P<0.01) associations with isolates of human origin were observed for *eae*, *stx*_2f_ and *ckf*. Highly significant (P<0.01) associations with isolates of non-human origin were observed for *stx*_2dact_ and *iha*. Other targets occurring in marginally significantly (0.01<P<0.05) higher frequency among isolates of human origin compared to isolates of non-human origin included *ent/espL2*, *nleA*, *nleG9*, *efa1*, *adfO*, and *nleH1–2* (Table 4).

**Table 4:** Summary of associations of gene targets with different groupings of isolates based on the Pearson Chi-square value (χ2) and the odds ratio (OR).

Target	HAS versus non-HAS		human versus non-human		*eae*-pos versus *eae*-neg		*eae*-neg human versus *eae*-neg non-human	
	χ^2^ ^a^	OR (95% CI) ^b^	χ^2^ ^a^	OR (95% CI) ^b^	χ^2^ ^a^	OR (95% CI) ^b^	χ^2^ ^a^	OR (95% CI) ^b^
*eae*	9.89**	4.0 (1.6–10.0)	9.04**	3.6 (1.5–5.66)				
*stx*_1_								
*stx*_2a_	7.68**	3.8 (1.4–10.5)			7.16**	3.2 (1.3–7.7)		
*stx*_2b_					16.38***	0.12 ^c^		
*stx*_2c_								
*stx*_2d_								
*stx*_2dact_			8.76**	0.17 (0.04–0.63)			6.5*	4.9 (1.3–18.6)
*stx*_2e_			4.49*	4.6^c^			5.65*	0.17
*stx*_2f_			8.60**	9.0^c^	47.96***	72.0 (9.0–575.7)		
*stx*_2g_								
*adfO*	20.16***	7.2 (2.8–18.7)	3.89*	2.5 (1.0–6.6)	124.8***	348 (43–2777)		
*astA*								
*chuA*								
*ckf*	16.97***	5.9 (2.4–14.8)	8.60**	3.7 (1.5–9.4)	96.63***	47 (18–125)	6.39*	0.15
*efa1*	31.57***	11.3 (4.2–30.1)	4.01*	2.8 (1.0–7.7)	108.4***	252 (32–1987)		
*ehxA*	14.78***	11.0 (2.5–48.1)						
*ent/espL2*	28.21***	11.3 (4.0–32.4)	4.47*	3.6 (1.0–12.9)	66.81***	62 (13–285)		
*etpD*					30.6***	35.8^c^	^d^	
*iha*	4.39*	3.1 (1.0–9.6)	6.79**	0.45 (0.25–0.83)	55.56***	17.6 (7.2–42.8)	3.92*	1.9 (1.0–3.8)
*iha_homologue*					4.38*	0.52 (0.26–1.05)		
*nleA*	13.00***	5.8 (2.0–16.7)			82.24***	152 (19–1194)		
*nleB*	9.96**	4.6 (1.7–13.0)			89.87***	92 (20–425)		
*nleB2*	3.90*	3.0 (1.0–9.0)			98.64***	187^c^	^d^	
*nleC*					30.6***	36^c^	^d^	
*nleD*					63.4***	91^c^	^d^	
*nleE*	17.83***	6.4 (2.5–16.4)			136.04***	443 (55–3562)		
*nleF*			3.89*	2.5 (1.0–6.6)	136.6***	394^c^	^d^	
*nleG*	9.35**	5.5 (1.7–18.1)			52.69***	81 (10–643)		
*nleG2–1*	6.47*	3.4 (1.3–9.3)			136.60***	394^c^	^d^	
*nleG2–3*	20.16***	7.2 (2.8–18.7)			136.6***	394^c^	^d^	
*nleG5–2*	31.96***	12.2 (4.4–34.1)			76.78***	75 (16–347)		
*nleG6–2*					35.15***	42^c^	^d^	
*nleG9*			3.89*	3.4 (0.9–12.1)	83.22***	138^c^	^d^	
*nleH1–1*	5.00*	3.4 (1.1–10.6)			88.30***	152^c^	^d^	
*nleH1–2*			4.47*	3.6 (1.0–12.9)	77.18***	137 (17–1081)		
*saa*					5.82*	0.17^c^		
*subA*					29.87***	0.03 (0.003–0.19)		
*terB*	6.89**	3.1 (1.3–7.6)			19.25***	4.7 (2.3–9.8)		
*toxB*	12.92***	9.6 (2.2–41.4)			35.15***	42.3^c^	^d^	
TspE4.C2								
*ureC*	16.79***	6.0 (2.4–15.5)						
*yjaA*	7.71**	0.13 (0.02–0.95)						

Empty cells mean no significant association.

^a^ Chi-square value. Asterisks indicate the level of significance

*** is P<0.001

** is 0.001<P<0.01

* is 0.01<P<0.05.

^b^ Odds-ratio (OR) and 95% confidence interval (CI).

^c^ One quadrant of the 2x2 contingency table contains a zero. For calculation of the odds-ratio the zero is replaced by 1. The given odds ratio value is the minimal value for that specific gene. No confidence interval is calculated.

^d^ No statistics were calculated because variable (gene) is a constant (i.e. does not occur among *eae*-negative isolates).

### Virulence factors differentiating HAS and non-HAS

In total, 20/40 virulence factors showed a significant higher association with HAS (Karmali SPT A, B and C) compared to non-HAS (Karmali SPT D and E) (Table 2). Highly significant (P<0.01) associations with HAS isolates were observed for (in decreasing order of strength of association): *nleG5–2*, *efa1*, *ent/espL2*, *ehxA*, *toxB*, *adfO*, *nleG2–3*, *nleE*, *cfk*, *ureC*, *nleA*, *nleG*, *nleB*, *eae*, *stx*_2a_, and *terB* (Table 4). Marginally significant associations (0.01P<0.05) with HAS isolates were observed for *nleH1–1*, *nleG2–1*, *iha*, and *nleB2* (OR 3.0).

### Virulence factors differentiating *eae*-positive and *eae-*negative non-O157 STEC

A significant higher number of virulence markers was observed among *eae-*positive (mean 9.6, median 7) compared to *eae-*negative isolates (mean 5.7, median 6) (P<0.001). All *eae*-negative strains lacked all 15 tested *nle*-genes, whereas the *eae*-positive strains on average showed 7.7 *nle*-genes (median 8.5). The virulence factors *stx*_2a_, *stx*_2f_, *ureC*, *terB*, *toxB*, *etpD*, *adfO*, and *cfk* showed a significant stronger association with *eae-*positive strains compared to *eae*-negative strains (Table 4). Several HAS were found negative for *eae* (mostly Karmali SPT-C): O76:H19 (n = 6), O128:H2 (n = 4) and O174:H21 (n = 4). The virulence factors *iha*_homologue, *saa*, *stx*_2b_, and *subA* showed a significant higher frequency among *eae*-negative strains. When comparing *eae-*negative non-human with clinical human STEC isolates, the factors *stx*_2dact_ (OR 4.9) and *iha* (OR 1.9) occurred in a significant higher frequency among *eae*-negative strains.

### Frequency of mutations in *rpoS*

Sequencing the *rpoS* gene revealed that 7/80 (8.8%) non-human isolates and 31/129 (24.0%) of the clinical isolates were characterized by mutations, including deletions, insertions and single nucleotide polymorphisms in the open reading frame (ORF). Surprisingly, the mutation found in the animal/meat isolates was nearly the same for all, i.e. A967G (N323D) (6/7). This mutation was also found in one human isolate. The majority of the *rpoS* mutated strains were phylogroup B1 (60.5%), followed by A (28.9%), D (10.5%) and B2 (none). With respect to the distribution of *rpoS* genotypes over seropathotypes, only one (1/15 = 6.7%) SPT-B isolate (O26:H11) was found with a mutated *rpoS*. Mutations were identified in 18/69 (26.1%) SPT-C isolates, 12/94 (12.8%) SPT-D isolates, and 6/31 (19.4%) SPT-E isolates. No relation was observed with the virulence profile.

## Discussion

The complexity regarding STEC in food safety enforcement as well as clinical care primarily relates to the current inability of designating individual strains as pathogens due to the large variety in serotype and genetic content associated with (severe) disease. Subsequently, pathogenicity can neither be excluded nor confirmed for a given STEC isolate based on the seropathotype concept or analysis of the public health surveillance data [7]. To classify the clinical and/or epidemic potential of a STEC isolate at an early stage is it crucial to identify virulence characteristics of putative pathogens from genomic information, which are referred to as ‘predictive hazard identification’ [7]. This study aimed at identifying associations between virulence factors, phylogenetic groups, isolation sources and seropathotypes in order to gain an increased understanding on the complex epidemiology of STEC.

### Most non-O157 STEC in the Netherlands are phylogroup B1 and associated with *ehxA*, *iha* and *stx*_2_, but not with *eae*

Consistent with previous studies [12,17], phylogenetic analysis shows that STEC are distributed over all four major phylogenetic groups but segregate mainly in phylogenetic group B1 and (to a lesser extent) A. This also reflects earlier observations concerning the broader host range, the more acute nature of infections, and the generally higher environmental persistence of B1 (and A) isolates compared to B2 and D isolates [18–21]. However, there is a relative paucity of information regarding the phylogenetic distribution of the virulence factors of STEC strains belonging to different phylogenetic groups. The observation by Girardeau *et al*. [12] that STEC isolates belonging to phylogroup A were exclusively *eae-*negative (and therefore “non-virulent”) could not be confirmed in the present study: i.e. 23.8% of the A isolates were *eae*-positive compared to only 12.9% of the B1 isolates. However, only phylogenetic group B1 and D contained HUS-associated strains (HAS). Possibly, these associations differ with respect to isolation sources and geographical regions. In contrast to intimin, the typical STEC virulence factors *stx*_2_ and *ehxA* were significantly associated with A and B1 isolates.

### The seropathotype is merely indicative for the virulence potential

Earlier studies showed a clear progressive decline in the number of *nle*-genes from SPT-A to SPT-E strains [8,22]. In the present study the relation between the SPT and the number of virulence factors was particular evident for the classical SPT classification and the *nle*-genes as compared to the modified EFSA classification [7] and the total number of virulence factors. The large variability in the number of virulence factors present among HAS indicates that this is merely indicative for the virulence potential. This is also evident from the variation within priority STEC serotypes, where the number of virulence factors present range from 7 to 25. Similarly, high numbers of virulence factor were observed among modified SPT-D en -E isolates which strikingly involved relatively many H- strains (O5:H-, O80:H-, O85:H-, O165:H-, O177:H-). The major problem with the SPT concept is that serogroups are retrospectively placed into risk classes. Given the amount of serogroups and large variation in genetic content this does not provide a proactive hazard identification system.

### The variation among STEC is characterized by correlated sets of virulence markers

STEC containing the LEE-island are characterized by their ability to express the attaching and effacing (A/E) phenotype, leading to substantial cytoskeletal rearrangements within the enterocyte [23]. Given the strong correlations between *eae* and other virulence markers, the disease mechanism employed by LEE-positive strains seems (unlike LEE-negative trains) strongly related to other virulence factors like *terB*, *toxB*, *etpD*, *adfO*, *ckf*, *efa1* (in random order) and almost all *nle*-factors. Primarily the isolates belonging to the EU top-5 serogroups possess this array of correlated virulence genes.

Although the LEE-island is considered a hallmark virulence factor for STEC pathogenesis, it appears not to be essential since sporadic cases and small outbreaks (including HC and HUS) have been caused by LEE-negative STEC [14,24]. Although mostly associated with less severe disease, 46% of all clinical human non-O157 STEC isolates in the Netherlands were *eae*-negative (Friesema, per. comm.). With the present study, the percentage of *eae*-negative human isolates was almost twice as high (80.9%). It has been postulated that in the absence of LEE-island mechanisms are emerging by which LEE-negative STEC interact with the host mucosa and induce disease [24]. The STEC autoagglutinating adhesion (*saa*), the iron-regulated gene homolog adhesion (*iha*) and the subtilase cytotoxin (*subAB*) have been reported as alternative adhesins [25–27]. In the present study, these three virulence factors indeed occurred in a significant higher frequency among LEE-negative strains. If and how the functions encoded by these virulence factors present in LEE-positive strains but lacking in LEE-negative strains are fulfilled should be a focus of further study.

Specific sets of virulence factors were also associated with different Stx-subtypes. Especially *stx*_2a_ was positively associated with an array of additional virulence factors (incl. *eae*) while *stx*_2b_, *stx*_2d_, *stx*_2e_ and *stx*_2g_ showed very little positive association with additional (known) virulence factors. Recently, the emergence of *stx*_2f-_STEC in the Netherlands was described, which are generally associated with more mild disease [28]. This might be explained by the relatively low potency of Stx2f [29], but also by the general absence of *ehxA* and *terB*, both showing a significant association with HAS in the current study and in general with EHEC/HUS [30,31]. These results highlight that differentiation in disease severity among different STEC is not likely linked to the presence or absence of a particular gene but to specific arrays of virulence factors (i.e. virulence profiles). The strong correlations between virulence factors indicate that these arrays are not a random collection but are rather specific sets. Comparative genomics of large sets of non-O157 STEC should reveal common genetic backbones and evolutionary processes leading to the acquisition of such sets of virulence factors [32].

### A large proportion of STEC isolates in the Netherlands are characterized by a relatively low risk virulence profile

In the Netherlands, the EU top-5 serogroups (O26, O103, O111, O145 and O157) represent approximately half of all clinical STEC isolates [13]. The other half is caused by serogroups O63 (10%), O91 (9%), O113 (6%), O146 (4%) and others. All evidence provided in the current study cumulates to the conclusion that isolates belonging to these serogroups are generally characterized by a low prevalence of virulence genes found to be associated with HAS in this study (like *adfO*, *cfk*, *eae*, *efa1*, *nle-genes*, *stx*_2a_, *terB*, *toxB*, and *ureC*; in alphabetic order). This coincides with the observation that most disease caused by these serogroups is relatively mild [13].

### Additional markers risk markers are needed to distinguish high risk from low risk STEC

In line with the results described here, Ju *et al*. [22] demonstrated that many of the non-LEE encoded effectors were primarily associated with *eae-*positive STEC strains. This is also supported by recent comparative genomics which revealed the absence of all known phage-encoded non-LEE effector genes *eae*-negative STEC [33]. Several ‘molecular risk assessment’ studies designated specific virulence profiles as strong signatures of high risk STEC. Bugarel *et al*. [34] concluded that the presence in the same strain of a core of virulence determinants of *eae*, *ent/espL2*, *nleB*, *nleE*, and *nleH1*–*2* is a strong signature of a human-pathogenic EHEC. A Belgian study presented the combined presence of *efa*, *nleE and stx*_2_ as a high-risk virulence profile [35]. Bosilevac *et al*. [36] reported the combination of *eae*, *nle* and *subA* genes as a high risk profile. However, all these markers were strongly correlated in the present study with *eae*, questioning the added value of using the *nle*-genes as an additional markers. Consequently, all *eae*-negative virulent STEC strains, including HAS [14] would be categorized as harmless STEC while other serotypes which have not been reported to be associated with severe disease or outbreaks but carry non-LEE-encoded virulence effectors similar to those of O157 EHEC, would be considered outbreak- and severe disease-associated serotypes. Therefore, we support the conclusion of Ju *et al*. [22] that additional markers or methods of assessment are needed to accurately distinguish highly pathogenic STEC from low-virulence or harmless STEC. This especially applies for *eae*-negative STEC. The factors *ehxA* and *ureC* were, independently from *eae*, associated with HAS. This indicates their candidacy as important pathogenicity markers next to *eae* and *stx*_2a_. The *ureC* was earlier identified as a suitable marker for pathogenicity [37]. Mutation of this gene resulted in reduced adherence of *E*. *coli* O157:H7 in ligated pig intestine [38] and strains with nonfunctional urease were less likely to survive stomach passage and colonize the mouse intestinal tract compared to urease positive strains [39]. However, in other studies *ureC* was strongly correlated to intimin [22,37]. Probably, different associations between virulence markers reflects a different STEC population composition in different geographical regions. Enterohemolysin (*ehxA*) is also is known for its association with HUS [30,31].

### *rpoS* variants are over-represented among human clinical isolates

Stationary-phase and almost any environmental stress that slows the growth rate of *E*. *coli* induce the *rpoS*-controlled general stress response [40]. In this study the frequency of isolates with *rpoS* variants (i.e. deviating from *E*. *coli* O157:H7 Sakai strain as wild-type (WT) reference) was three times higher among human clinical isolates compared to animal and food isolates. A similar skewed distribution of WT and variants was demonstrated for STEC O157 isolates [15]. Th variants were negatively associated with survival in soil and resistance to acid shock. The postulated hypothesis by van Hoek *et al*. [15] on the human gut as an environment that would give rise to *rpoS* variants is strengthened by the current results on non-O157. There is evidence that a WT functional *rpoS* is advantageous for bovine colonization [41,42]. In contrast, *rpoS* negatively regulates the expression of the LEE-island [43] and negatively affects the colonization of mice [44]. Non-bovine enteric systems could select for *rpoS* variants as these are characterized by increased nutrient scavenging abilities at the expense of stress-resistance [45]. Hereby, direct competition with commensal *E*. *coli* could be avoided by the establishment of an STEC specific metabolic niche [46].

## Conclusions

The large variability in the number of virulence factors present among serogroups and seropathotypes indicated that this was merely indicative for the virulence potential. While all the virulence gene associations have been compared, it appeared that there is no specific pattern that would unambiguously enable hazard identification for an STEC strain. However, the strong correlations between virulence factors indicate that these arrays are not a random collection but are rather specific sets. Most non-O157 STEC in the Netherlands are phylogroup B1 and characterized by the presence of *ehxA*, *iha and stx*_2_, but absence of *eae*. Especially *eae* was strongly correlated to many of the other virulence genes, including all non-LEE encoded effectors. Different *stx-*subtypes were associated with different virulence profiles. The factors *ehxA* and *ureC* were independently from *eae* associated with HUS-associated strains (HAS). This indicates their candidacy as important pathogenicity markers next to *eae* and *stx*_2a_. However, since some serogroups are only represented by a limited number of isolates definitive conclusions on association of virulence factors with these groups require a more specific strain set.

## Supporting Information

S1 Table Characteristics and typing results of all strains. (XLSX)
